# Obstetric and psychological characteristics of women choosing epidural analgesia during labour: A cohort study

**DOI:** 10.1371/journal.pone.0186564

**Published:** 2017-10-18

**Authors:** Vasilis Sitras, Jūratė Šaltytė Benth, Malin Eberhard-Gran

**Affiliations:** 1 Department of Obstetrics and Gynaecology, Akershus University Hospital, Oslo, Norway; 2 Department of Fetal Medicine, Oslo University Hospital, Oslo, Norway; 3 Institute of Clinical Medicine, University of Oslo, Oslo, Norway; 4 Health Services Research Unit, Akershus University Hospital, Lørenskog, Norway; 5 Department of Child Health, Norwegian Institute of Public Health, Oslo, Norway; National Academy of Medical Sciences, NEPAL

## Abstract

**Objectives:**

To investigate the obstetric and psychological characteristics of women who opt to use epidural analgesia (EDA) during labour and the impact of participating in labour preparation courses on women’s decisions to use EDA.

**Design:**

Longitudinal cohort study.

**Setting:**

Akershus University Hospital, Norway.

**Population:**

2596 women with singleton pregnancies and intended vaginal delivery.

**Methods:**

Data were collected using two self-completed questionnaires at pregnancy weeks 17 and 32. Fear of childbirth was assessed by the Wijma Delivery Expectancy Questionnaire (W-DEQ). Symptoms of anxiety were measured by the Hopkins Symptom Check List (SCL-25) and depression by the Edinburgh Postnatal Depression Scale (EPDS). Obstetric and socio-demographic information was retrieved from birth records at the maternity ward.

**Main outcome measure:**

Preference for EDA was indicated by the questionnaire item “I would prefer an epidural regardless” on a 4-point scale (1 = highly agree, 4 = highly disagree) at pregnancy week 32.

**Results:**

Twenty-one percent of the women (540/2596) answered that they would choose EDA as the only alternative method of analgesia during labour. Counselling for fear of childbirth [OR 3.23 (95%CI 2.12; 4.92)] and W-DEQ sum score ≥ 85 [OR 2.95 (95%CI 2.06; 4.23)] were significantly (p<0.001) associated with choice of EDA. Participation in labour preparation courses was significantly (p = 0.008) associated with a reduction of intended use of EDA during labour [OR 0.67 (95%CI 0.49; 0.90)].

**Conclusion:**

Fear of childbirth is significantly associated with women’s choice of EDA during labour. On the other hand, women that participate in labour preparation courses would rather consider other methods of analgesia during labour.

## Introduction

Associated with actual or potential tissue damage, pain is a distressing experience involving sensory, emotional, cognitive and social components.[1] A woman’s experience with pain during labour varies widely due to social, psychological and physiological factors. It is reasonable to believe that previous childbirth experience and childbirth self-efficacy, as well as symptoms of fear and anxiety influence a woman’s choice of pain relief during labour. Given such diversity of experiences with childbirth, a recent Cochrane Review concluded that “Women should feel free to choose whatever pain management they feel would help them most during labour”.[2]

Among means of labour pain management, epidural analgesia (EDA) is a common, effective pharmacological intervention that both mothers and foetuses generally tolerate well. Accordingly, in the absence of maternal medical contraindications such as coagulopathy, hypovolemia, infection at the puncture site, and high intracranial pressure, EDA should be offered to all parturient women. However, side effects of EDA include the increased need for instrumental vaginal delivery, maternal hypotension, motor block, fever, and urine retention.[3] Moreover, mothers with hypotension can experience foetal distress due to reduced foetal-placental perfusion, and far more rarely, the accidental intrathecal injection of anaesthetic medication can cause high spinal blockade, respiratory complications, seizure, and cardiac arrest. For these reasons, parturient women using EDA, their foetuses, and once born, their infants are monitored closely throughout labour and immediately postpartum. Alternative methods of pain relief during labour include self-help (coping), water birth, acupuncture, inhalation of nitrous oxide gas, sterile water blocks and pethidine injections. For women who manage to cope with labour pain themselves or with the psychological support of their partners, medical or alternative methods of pain relief are not necessary. For women who need pain relief, EDA has the advantage of continuous and efficient analgesia compared to all other methods that provide less pain relief or are used as adjunctive to self-coping. Since the majority of women decide in advance, whether they should use EDA or not [4] all these aspects of EDA should be discussed antenatally, helping parturient women make informed choices regarding pain relief during childbirth. It has therefore been speculated whether participation in labour preparation courses affects women’s preferences for pain relief during labour.[5]

Several studies have investigated factors influencing women’s decisions to use EDA during labour, [6–9] although only two [4,10] focused on the impact of psychological factors. These two studies were, however, limited by small sample sizes or use of non-validated questionnaires.[10] The objectives of this study were to investigate possible obstetric and psychological factors associated with EDA preference during labour and the impact of participating in labour preparation courses on women’s decisions to use EDA.

## Materials and methods

### Participants

**Fig 1:**
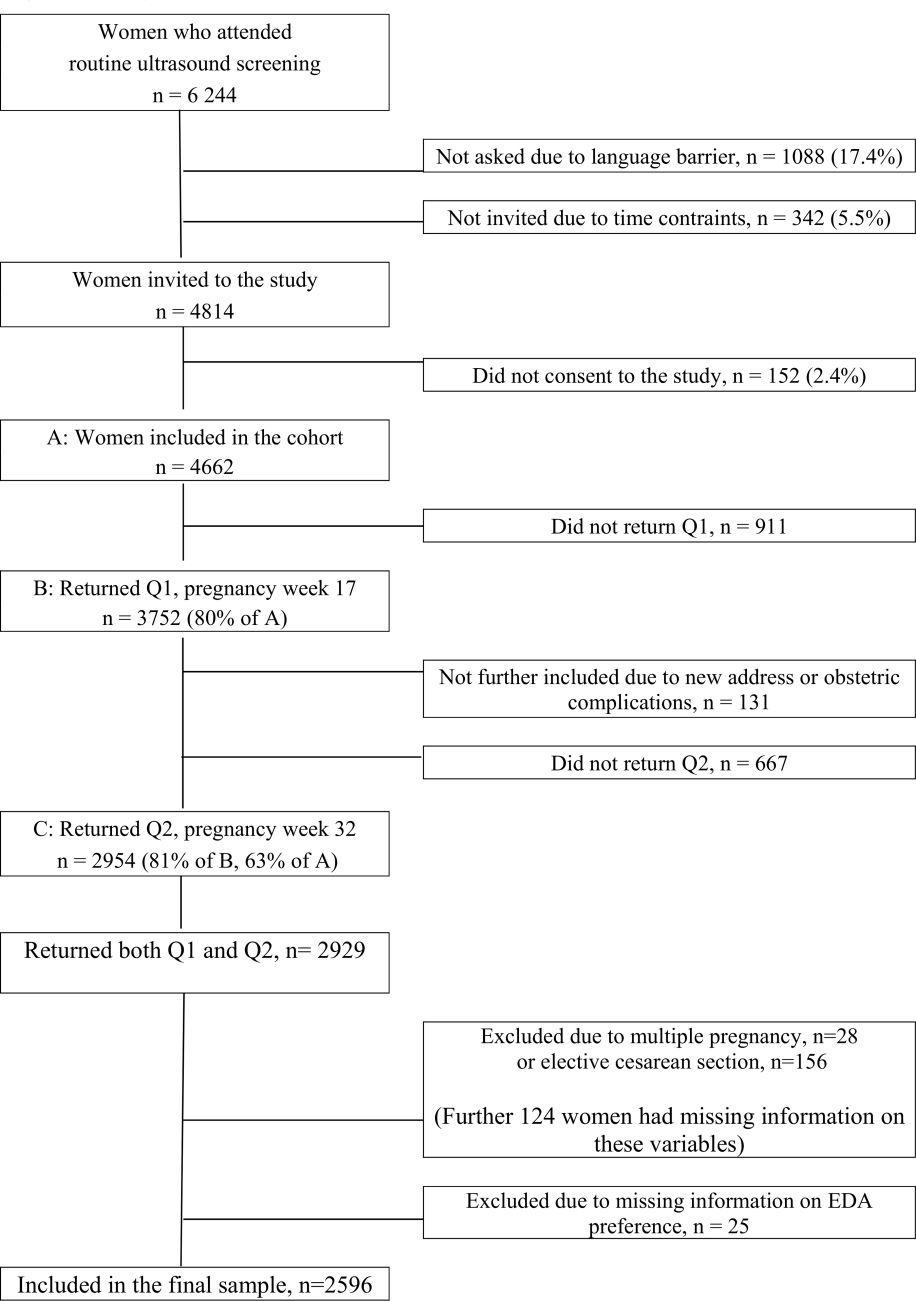
Flow chart displaying the recruitment and retention of the participants used in the present study.

The sample formed part of the Akershus Birth Cohort Study, which is described in detail elsewere [11,12]. Fig 1 displays a flow-chart of the recruitment and retention of participants used in the present study.

In total, 2929 women returned both questionnaires and comprised our baseline sample. Additional information on the pregnancies and births was obtained by linkage to the electronic birth records at the obstetric ward. The doctor or midwife in charge of the delivery completed the birth records. We excluded women with multiple pregnancies (n = 28) or planned caesarean section (n = 156). We further excluded women with missing information on these two parameters (n = 124), in addition to missing information regarding EDA preference (n = 25). This resulted in a final sample of 2596 women.

### Ethics approval

All women asked to participate received written information explaining the purpose of the study and that their participation was voluntary. Written informed consent was obtained from all participants. The study was approved by the Regional Committee for Ethics in Medical Research in Norway (approval no. S-08013a).

### Measures

At 32 weeks gestation, participants indicated their preference for EDA by responding to the questionnaire item “I would prefer an epidural regardless” on a 4-point scale (1 = highly agree, 4 = highly disagree). Answers were coded as; yes (i.e. Highly agree or Agree) or no (i.e. Disagree or Highly disagree).

Fear of childbirth was assessed with the Wijma Delivery Expectancy/Experience Questionnaire version A (W-DEQ), a 33-item self-assessment rating scale, responses to which are rated on a 6-point Likert scale from 0 to 5.[13] Sum score ranges from 0 to 165, with higher scores reflecting a greater degree of fear of childbirth. In the data analyses, fear of childbirth was defined as a W-DEQ total score ≥85. This cut-off has been commonly used to distinguish between women with and without fear of childbirth.[14] Details regarding the Norwegian version of the W-DEQ are described elsewhere.[15]

Participants indicated their attendance of labour preparation courses by answering the item “I have attended a labour preparation course”. The aims of these courses are to increase knowledge about physiology of pregnancy and childbirth in order to enhance the feelings of safety and self-control during pregnancy and labour. Women with depression and/or anxiety related to childbirth were followed by dedicated midwifes and/or obstetricians throughout pregnancy in our hospital. Therefore, questionnaires also gathered whether women had received counselling for pregnancy concern (including self-reported fear of childbirth) by affirming or disaffirming the item “Do you receive counselling for pregnancy concern (fear of childbirth)?”

Anticipated pain during the upcoming labour was measured in pregnancy week 32 using a numeric rating scale (NRS) and based on the question “How much pain do you think you will feel during labour?” with scores from 1 (No pain) to 10 (Greatest pain imaginable).

We hypothesized that pre-pregnancy menstrual pain might be associated with a woman’s perception of pain in the lumbar region and/or genital organs. Pre-pregnancy menstrual pain was measured using an NRS and based on the following question in the first questionnaire “How much pain do you normally experience during menstruation?” with scores ranging from 1 to 10. Participants addressed pelvic girdle pain by answering affirmatively or negatively to the questions “Do you frequently wake up at night because of pelvic girdle pain?” and “Do you use crutches because of pelvic girdle pain?”

Having symptoms of depression or anxiety was defined as having a score ≥ 13 on the 10-item self-rated Edinburgh Postnatal Depression Scale (EPDS) [16] and/or a score ≥ 18 on the first 10 items (SCL-anxiety) of the 25-item Hopkins Symptoms Checklist (SCL-25),[17,18] in either questionnaire at 17 or at 32 weeks gestation. Both instruments are widely used and validated as tools for detecting symptoms of depression and anxiety in pregnancy. [19–20]

Information regarding medical risk factors was retrieved from birth records at the maternity ward of Akershus University Hospital. Each risk factor was treated as a dichotomous variable, depending on whether it appeared during pregnancy. Risk factors included heart disease, chronic or pregnancy induced hypertension, chronic kidney disease, asthma, epilepsy, rheumatoid arthritis, diabetes, and preeclampsia before 34 weeks of gestation and were coded as none or ≥ 1 risk factor(s). Information concerning maternal education and age at delivery was obtained from birth records at the maternity ward as well. Years of education of mothers was coded as ≤12 or >12. Participants reported parity in the first questionnaire, which was coded nulliparous (parity = 0) or multiparous (parity≥1).

### Statistical analyses

Variables were described as means and standard deviations (SD) or as frequencies and percentages among participants intending to choose EDA and those without such intention. Bivariate logistic regression models were estimated for EDA for each pre-specified predictor and confounder, after which a multivariate logistic regression model with all considered predictors and confounders was estimated. Interactions between parity and each predictor were entered into the multivariate model to assess potential differences in predictors for EDA between nulliparous and multiparous women. The multivariate model was reduced by applying Akaike’s Information Criterion, in which a lesser value indicates a better model. Results were presented as crude and adjusted odds ratios (OR) with corresponding 95% confidence intervals (CI) and p-values. Regression coefficients and standard errors (SE) were reported for variables included in the interaction terms. All tests were two-sided. Results with p-values below 0.05 were considered to be statistically significant. All analyses were performed in the Statistical Package for the Social Sciences version 24.

## Results

Twenty-one percent of the women (540/2596) would choose EDA as the only alternative method of analgesia during labour. The mean maternal age was 30.9 years (range 18–45, SD = 4.7). Fifty-one percent of the women were nulliparous. The majority of the participants (n = 1637, 63.1%) had higher education. Twenty-two percent of the women (561/2596) had at least one medical risk factor. Nineteen percent of the women (495/2596) had attended labour preparation courses (Table 1).

**Table 1:** Characteristics of the sample according to whether the participants opt to use epidural analgesia (EDA) during labour or not.

Variable	No EDA preference	EDA preference	Total
	n (% or ±SD)	n (% or ±SD)	n
*Maternal age (years)*	30.9 (±4.7)	31.0 (±4.8)	30.9 (±4.7)
*Educational level (years)*			
≤12	634 (73.4)	230 (26.6)	864
>12	1348 (82.3)	289 (17.7)	1637
Missing			95
*Parity*			
Nulliparous	1050 (79.7)	267 (20.3)	1317
Multiparous	1006 (78.7)	273 (21.3)	1279
*Fear of childbirth (W-DEQ)*			
Low score (<85)	1935 (81.5)	439 (18.5)	2374
High score (≥85)	95 (52.2)	87 (47.8)	182
Missing			40
*Consultation for pregnancy concern*			
No	1998 (80.7)	478 (19.3)	2476
Yes	58 (48.3)	62 (51.7)	120
*Medical risk factors*			
None	1649 (81.0)	386 (19.0)	2035
One or more	407 (72.5)	154 (27.5)	561
*Labour preparation course*			
No	1583 (78.3)	440 (21.7)	2023
Yes	414 (83.6)	81 (16.4)	495
Missing			78
*Pelvic girdle pain*			
No	1995 (79.1)	526 (20.9)	2521
Yes	61 (81.3)	14 (18.7)	75
*Mental Health*			
No mental impairment	1831 (80.6)	442 (19.4)	2273
Depression and/or anxiety	219 (69.3)	97 (30.7)	316
Missing			7
*Anticipated pain during labour (NRS)*	7.6 (±1.9)	8.4 (±1.7)	7.7 (±1.8)
Missing			13
*Pre-pregnancy menstrual pain (NRS)*	3.4 (±2.3)	3.8 (±2.4)	3.5 (±2.3)
Missing			9

### Factors associated with a preference for EDA

A preference for EDA was observed among 51.7% (62/120) of the women who received consultation for pregnancy concern, and 47.8% (87/182) of women who scored above the threshold on the W-DEQ. According to binary logistic regression models, we found that consultation for pregnancy concern [crude OR 4.6, 95% CI 3.1–6.8)] and a high score on the W-DEQ [crude OR 4.2 (95% CI 3.0–5.8] were highly associated with preference for EDA. In the multivariate regression model, consultation for pregnancy concern remained strongly associated with preference for EDA (aOR 3.2, 95% CI 2.1–4.9), followed by a high score on the W-DEQ (aOR 3.0, 95% CI 2.1–4.2). Participation in labour preparation courses was significantly (p = 0.008) associated with a reduction of intended use of EDA during labour [aOR 0.7 (95% CI 0.5; 0.9)] (Table 2).

**Table 2:** Unadjusted and adjusted odds ratios (OR) with 95% confidence intervals (CI) for preference for epidural analgesia (EDA).

Variable	Bivariate analysis		Multivariate analysis	
	OR (95% CI)	P-value	OR (95% CI)	P-value
Fear of childbirth	4.16 (3.01; 5.75)	**<0.001**	2.95 (2.06; 4.23)	**<0.001**
Anticipated pain during upcoming birth	1.28 (1.20; 1.36)	**<0.001**	1.22 (1.14; 1.30)	**<0.001**
Consultation for pregnancy concern	4.58 (3.11; 6.75)	**<0.001**	3.23 (2.12; 4.92)	**<0.001**
Labour preparation course	0.73 (0.56; 0.95)	**0.018**	0.67 (0.49; 0.90)	**0.008**
Pre-pregnancy menstrual pain	1.08 (1.03; 1.13)	**0.001**	1.04 (0.997;1.09)	0.068
Medical risk factors	1.62 (1.29; 2.03)	**<0.001**	-^2^	-
Pelvic girdle pain	0.97 (0.53; 1.75)	0.910	0.61 (0.32; 1.16)	0.134
Mental Health	1.95 (1.49; 2.56)	**<0.001**	-0.01 (0.23)^1^	0.966
Mental Health x Parity	-	**-**	0.51 (0.30)^1^	0.092
Parity	1.06 (0.87; 1.29)	0.587	-0.23 (0.13)^1^	0.080
Maternal age (continuous variable)	1.00 (0.98;1.02)	0.824	1.02 (0.997;1.05)	0.080
Educational level (basic = 0, higher = 1)	0.60 (0.49; 0.73)	<0.001	0.56 (0.45; 0.70)	<0.001

^1^ Coefficient (SE) presented instead of OR (95% CI) due to interaction term between Mental Health and Parity; see Table 3 below for interpretation

^2^ Akaike’s Information Criterion (AIC) suggests that “Medical risk factors” can be eliminated from the model

Even though not significant, the interaction term between “parity” and “mental health” was the only one left in the multivariate model after applying AIC. Exploring the interaction term further showed that multiparous women with mental health problems had 65% higher odds for choosing EDA during labour compared to multiparous women without mental health problems (OR 1.65 (95% CI 1.10; 2.47), p = 0.016) (Table 3).

**Table 3:** Interpreting the interaction between “mental health” and “parity”.

Mental Health	Nulliparous	Multiparous	OR for nulliparous	
	Odds for EDA (95% CI)	Odds for EDA (95% CI)	OR (95% CI)	P-value
No mental impairment	0.05 (0.02; 0.16)	0.04 (0.01; 0.14)	1.26(0.97;1.63)	0.080
Depression and/or anxiety	0.05 (0.02; 0.17)	0.07 (0.02; 0.24)	0.76(0.43;1.33)	0.338
OR for Mental Health = 1 (High/High)				
OR (95% CI)	0.99 (0.63; 1.55)	1.65 (1.10; 2.47)		
p-value	0.966	**0.016**		

## Discussion

In this large cohort study, we investigated several obstetric and psychological factors characterizing women’s choice for pain relief measures during labor. We found that women with fear of childbirth would choose EDA, whereas women who participated in labour preparation courses would rather consider other methods for pain relief during labour. Moreover, multiparous women with mental health problems had increased odds for choosing an EDA, compared to multiparous women without mental health problems.

This study marks one of the few large cohort studies to not only examine psychological factors characterizing differences between women with and without an EDA preference, but also to investigate the impact of participation in labour preparation courses on women’s decisions to use EDA. To our knowledge, this is the first large cohort study examining the linkage between maternal psychological characteristics and an EDA preference, which took such a large number of socio-demographic, mental and somatic risk factors into account.

The study enjoyed a high participation rate (80%) and included women recruited at routine examinations, indicating that selection bias was low. Moreover, given this study’s access to medical records and maternity ward birth records, information regarding mode of delivery and medical risk factors supported the prospective design. Furthermore, in contrast to other studies fear of childbirth was measured with a validated instrument designed to measure fear of childbirth, the W-DEQ.[13,20] Moreover, EPDS and SCL-anxiety are validated screening instruments used to identify women with probable depression and anxiety.[21,22]

Still, some potential limitations are worth discussing. No established, validated instrument is currently available for measuring preference for EDA or anticipated pain. Consequently, we used a numeric one-item scale shown to be reliable and valid for measurements of pain, mood, and other subjective feelings.[23] There is reason to believe that the women in the study were somewhat more resourceful than the general birthing population in Norway. There were fewer younger women (13 vs. 17%) and fewer single women (4 vs. 7%) compared with national data obtained from the Medical Birth Registry of Norway. However, it is unlikely that this possibly skewed selection could have biased the estimated directions of associations between maternal psychological characteristics and EDA preference. Nevertheless, the generalizability of the results of this study may be limited by the fact that only Norwegian-speaking women were included, which resulted in a relatively homogenous, almost entirely Caucasian sample. Lastly, we decided to control for a list of variables that were available in our dataset and that we believed might be important for the outcome. Other confounders that we did not measure could possibly play a role.

According to the Norwegian Medical Birth Registry, EDA is the main method for pain relief during labour and its use has gradually increased in Norway from 27% in 2005 to 33% in 2014 (http://statistikk.fhi.no/mfr/) (S1 Fig). In Norway deliveries occur solely in public hospitals and all parturient women have free access to obstetric care, including obstetric anaesthesia service. A recent randomised controlled trial has shown that the cost of epidural analgesia at request is comparable with the cost of EDA performed routinely to all parturient women.[24] However, routine EDA was associated with more EDA-related maternal adverse effects (hypotension and motor blockade) and more operative deliveries. These facts depict the importance of characterising better the group of women who choose EDA as the only method of analgesia during labour, aiming to better inform them about the possible adverse outcomes for them and their babies related to EDA.

Our study showed that women participating in labour preparation courses would rather not choose EDA during labour. This result is in contrast with a Swedish national cohort study performed in 1999, indicating that women who attended childbirth classes had higher rates of EDA.[5] The authors proposed that participation in classes increased awareness of pain relief techniques available, rather than improving women’s coping with pain. Moreover, younger mothers, with low level of education, living in smaller cities were less likely to find the classes helpful. They concluded that the current form of antenatal education in Sweden might not be effective. In the contrary, our study was performed recently, in a large hospital and the majority of participating women had higher education. Furthermore, a purpose of the preparation courses in our hospital was to inform coming mothers about the physiology of pregnancy, labour and puerperium, giving emphasis on the woman’s own ability to cope with pain and improve the experience of childbirth. Specific focus was pointed towards labour pain, other methods of pain relief and towards the medical contraindications, precautions and adverse side effects of EDA. Hence, we hypothesize that women who participated in pregnancy education program were made aware of and reflected on the fact that labour pain is a physiological mechanism during delivery that is self-limiting (disappears after birth) and is generally well tolerated with other methods of pain relief.

## Conclusions

Fear of childbirth is significantly associated with women’s choice of EDA during labour. On the other hand, women that participate in labour preparation courses would rather consider other methods of analgesia during labour. We propose that more efforts should be undertaken from health practitioners (general physicians, midwifes and obstetricians) to inform coming mothers about the physiology of childbirth and possible methods of pain relief during labour.

## Supporting information

S1 Fig Use of epidural analgesia per 1000 deliveries according to the Norwegian Medical Birth Registry during the period 2005–2014. (PNG)
