# Supplementary figures and images for: Obstetric and psychological characteristics of women choosing epidural analgesia during labour: A cohort study
Source: PLoS One. 2017 Oct 18;12(10):e0186564. doi: 10.1371/journal.pone.0186564 (PMC5646833; doi:10.1371/journal.pone.0186564)

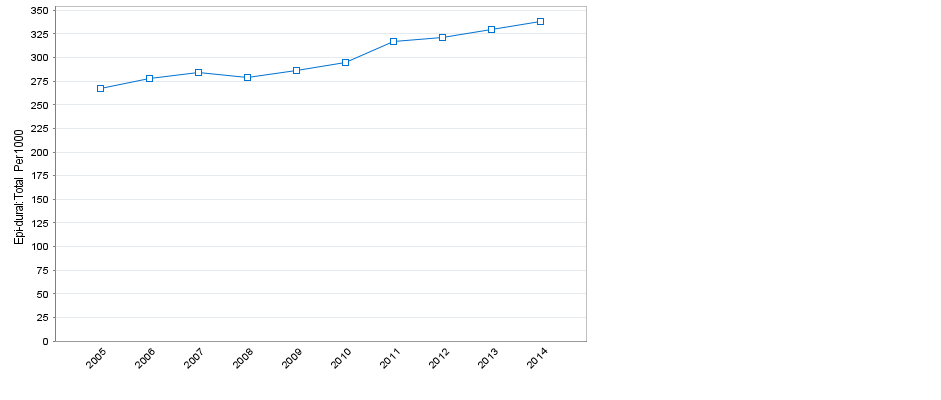

Supplement: S1 Fig — (PNG) [file pone.0186564.s001.png]
